# Extracellular vesicles in glioblastoma: a challenge and an opportunity

**DOI:** 10.1038/s41698-024-00600-2

**Published:** 2024-05-17

**Authors:** Vineesh Indira Chandran, Srinivas Gopala, Easwer Hariharan Venkat, Mads Kjolby, Peter Nejsum

**Affiliations:** 1 Department of Clinical Medicine, Aarhus University, Aarhus, Denmark; 2 Department of Infectious Diseases, Aarhus University Hospital, Aarhus, Denmark; 3 Department of Biochemistry, Sree Chitra Tirunal Institute for Medical Sciences and Technology, Thiruvananthapuram, Kerala India; 4 Department of Neurosurgery, Sree Chitra Tirunal Institute for Medical Sciences and Technology, Thiruvananthapuram, Kerala India; 5 Department of Biomedicine, Aarhus University, Aarhus, Denmark; 6 Department of Clinical Pharmacology and Steno Diabetes Centre, Aarhus University Hospital, Aarhus, Denmark

**Keywords:** CNS cancer, Extracellular signalling molecules

## Abstract

Glioblastoma is a highly heterogeneous tumor whose pathophysiological complexities dictate both the diagnosis of disease severity as well as response to therapy. Conventional diagnostic tools and standard treatment regimens have only managed to achieve limited success in the management of patients suspected of glioblastoma. Extracellular vesicles are an emerging liquid biopsy tool that has shown great promise in resolving the limitations presented by the heterogeneous nature of glioblastoma. Here we discuss the contrasting yet interdependent dual role of extracellular vesicles as communication agents that contribute to the progression of glioblastoma by creating a heterogeneous microenvironment and as a liquid biopsy tool providing an opportunity to accurately identify the disease severity and progression.

## Introduction

Primary brain tumors or generally called gliomas are among the most commonly occurring and difficult-to-treat cancers of the central nervous system. They are most common in adults and comprises almost 80% of all malignant primary tumors of the brain^1^. Gliomas are heterogeneous glial cell-derived malignancies that primarily develop as diffuse tumors and extensively infiltrate the brain parenchyma^2^. The fifth WHO update released in 2021 organized the degree of malignancy of adult-type diffuse gliomas into astrocytoma, IDH-mutant; oligodendroglioma, IDH-mutant and 1p/19q-codeleted; and glioblastoma, IDH-wildtype^3^. Among the glioma subtypes, IDH-wildtype glioblastomas are the most common and aggressive, having one of the worst overall prognoses with a best 5-year survival rate of less than 10 percent^4,5^. The current standard of care for patients with glioblastoma is surgery followed by radiotherapy with concurrent or adjuvant chemotherapy. Despite multimodal treatment strategies, glioblastoma patients have a median survival time of 12–15 months^6–8^. Therefore, introduction of novel immunotherapeutic approaches was expected to improve the glioblastoma treatment scenario substantially and reduce the high rates of disease relapse. However, glioblastoma patients still develop resistance despite recent advances in immunotherapy^9^.

Meanwhile, accurate and timely diagnosis is key to improved survival in glioblastoma patients. Currently, glioblastoma is diagnosed by imaging techniques and invasive tissue biopsies^10^. Non-invasive imaging techniques are unreliable as they cannot definitively differentiate lesions caused by actual tumor progression from pseudoprogression. Tissue biopsies, on the other hand, entail a highly invasive procedure, yet might only capture a static snapshot of the progressive tumor^11^. Most of these therapeutic setbacks and diagnostic limitations can be attributed to the extent of heterogeneity within glioblastoma and their associated microenvironment that collectively impacts the ability to identify and treat the disease accurately^12^.

In this review, we discuss heterogeneity in glioblastoma and provide a detailed account of extracellular vesicles (EVs) as one of the major actors in glioblastoma heterogeneity. Then, we review the heterogeneity in EVs released by glioblastoma cells and the role of heterogeneous EV-mediated symbiotic and reciprocal communication between tumor cells and other stromal cells in creating and fostering a highly heterogeneous tumor microenvironment (TME). Further, we make a strong case for EVs that as one of the main actors involved in creating a challenging pro-tumorigenic microenvironment, can be ideal for the stratification of glioblastoma subtypes as well as constant monitoring of disease progression. Finally, we propose novel short-term and long-term strategies based on EV biomarkers, which can either supplement current diagnostic methods or replace existing life-threatening invasive procedures used for glioblastoma diagnosis and evaluation of treatment response.

## Glioblastoma heterogeneity

Glioblastomas are aggressive, rapidly proliferating tumors that are characterized by increased mitotic activity, high invasiveness, and areas of necrosis^13^. One of the hallmarks of glioblastoma is the high degree of heterogeneity determined by various tumor-intrinsic and extrinsic drivers^14^. Glioblastoma heterogeneity is characterized by the presence of clonal and subclonal differentiated cell populations, glioma stem cells (GSCs) and other components of the tumor microenvironment^15^. Intrinsic mechanisms that drive intra-tumoral heterogeneity include the host of highly mutated genome with several tumor-promoting genetic and epigenetic modifications besides the nature of the cell of origin. Whereas inter-tumoral heterogeneity is imposed by extrinsic factors from the TME that are mediated by the interaction of glioma cells with the components of their associated TME. Heterogeneity within glioblastoma with vast diversity of cell states, composition of cells, and phenotypical characteristics enable glioma cells to adapt their identity towards a pro-tumorigenic paradigm and escape treatment^16–18^.

Extensive profiling of genetic and mutational landscape in combination with transcriptional profiles have identified different subtypes of glioblastoma. These glioblastoma subtypes are either distinguished by the expression levels of *EGFR*, *NF1*, and *PDGFRA/IDH1* or characterized by the status of overall chromatin accessibility, giving them varied sensitivities to drug treatment^19,20^. More importantly, during glioblastoma progression and recurrence, they may exhibit high plasticity and intra-convert to a different subtype^21^ that poses a significant therapeutic challenge. Upon applying single-cell RNA-sequencing technologies, researchers gained more clarity into the plasticity of glioblastoma subtypes in enhancing intra-tumoral heterogeneity. Further analyses of spatially distinct tumor fragments revealed that patients not only displayed different subtypes within one tumor but also harbored different copy number alterations in genes such as *EGFR*, *PTEN*, and *PDGFR*, ultimately resulting in several cell lineages coexisting within the same tumor^22,23^.

Among the existence of multiple glioma cell types, even though the evolution and functions of GSCs in glioblastoma are controversial, their presence is largely undisputed. This is mainly due to the increased prevalence of resistance to radio- and chemotherapy in glioblastoma patients, which is largely attributed to the interactions of GSCs in shaping a heterogeneous TME^17,24^. Therefore, it is not surprising that GSCs are implicated in the maintenance of residual tumor leading to recurrent disease as well as development of new lesions^25^. While a direct role for GSCs in cultivating heterogeneity at new metastatic lesions is yet to be established, a longitudinal analysis of IDH-mutant astrocytomas revealed significant difference in the GSC-derived tumor-associated microglia/macrophages transcriptional state between oligodendrogliomas and astrocytomas during recurrence^26^.

Meanwhile, in addition to copy number variations^27^, therapeutic intervention can result in complex changes in the glioblastoma landscape contributing to heterogeneity in glioma cell states in different patients^28^. Single cell RNA sequencing studies reflected on this inter-patient heterogeneity by unearthing diversity in specific populations of immune cells in the TME^29^. For example, treatment of glioblastoma patients with anti-PD-1 showed an enrichment of *PTEN* mutations with an associated increase in immunosuppressive signatures in non-responders compared to responders^30^. Limited success of promising immune checkpoint inhibitor approaches including anti-PD-1 in glioblastoma can be attributed to these dynamic changes in the immune TME^31^. Taken altogether, cumulative evidence shows that heterogeneity strongly differs between new and recurrent as well as treatment naïve and experienced glioblastoma patients in terms of molecular characterization, interpretation of tumor prognosis and response to treatment, and monitoring of tumor progression^32^. A detailed account of intra-tumoral, and inter-tumoral heterogeneity in glioblastoma can be found elsewhere^33^. Moreover, inter-observer variations in histopathological diagnosis further amplifies the complexities associated with glioblastoma heterogeneity^34^. All of these present an additional limitation to finding and establishing unified treatment approaches and calls for adopting liquid biopsy-based tools for more individualized treatment plans.

## Glioblastoma-derived extracellular vesicles

EVs are lipid-bilayer bound nanoparticles (30–1000 nm in size) that are released by all cell types and can be found in all biological fluids. EVs carry a wide variety of proteins, lipids, nucleic acids, and other bioactive molecules depending on their host cell of origin. Although EVs were initially considered to be a reservoir that eliminates waste from cells, the well investigated narrative now looks beyond their characterization as mere waste carriers and focuses on the capacity to exchange bioactive molecular components between cells^35^. The latter function has generated enormous interest within the scientific community to investigate the purported role of EVs in different pathophysiological contexts. In fact, the critical nature of their engagement with other cells in pathological as well as normal physiological processes has directly been linked to the EV cargo^36,37^. Interestingly, the cargo carried by EVs that are released from different cells are not homogeneous in nature. From any given source, the population of EVs comprises of diverse subpopulations which can differ in composition, size, morphology, and/or mechanisms of biogenesis^35^.

Depending on the size, EVs are mainly classified into exosomes (30–120 nm), microvesicles (50–1000 nm), apoptotic bodies (50–2000 nm), and large oncosomes (1–10 µm). These vesicles also have different biogenic mechanisms where exosomes are formed as intraluminal vesicles within multivesicular bodies (MVBs), that are released by cells upon fusion of MVBs with the plasma membrane. On the other hand, large oncosomes and microvesicles including ectosomes, shedding vesicles, and microparticles originate directly from the plasma membrane. Apoptotic bodies are also categorized as vesicular structures that form by outward blebbing and fragmentation of membraneous parts from cells undergoing apoptosis^38^. A detailed account of different types of EVs and the machineries involved in their biogenesis is discussed elsewhere^35,39^. The different biogenic mechanisms play a major role in the selective packaging of cargo into these EVs, further enhancing their heterogeneous profile. One of the early studies that demonstrated heterogeneity in EV cargo looked at the differential packaging of miRNAs into distinct EV subpopulations released by tumor cells^40^. Since then, several studies have come out with notable findings on loading of different molecular components in EVs with respect to specific functional properties^41^. In addition to the above factors, the overall heterogeneity of EVs also reflect the prevalent dynamic changes in cellular differentiation status^42^. For instance, Saito et al.^42^, found that changes in EV miRNA and protein expression was reflective of the differentiation status of induced pluripotent stem cells (iPSCs). The authors specifically observed the expression of miR-106b in EVs, that is involved in neural stem cell differentiation, to change significantly between iPSCs and differentiated cells. Taken altogether, cumulative findings have led to an evidence-based consensus for not only specific and controlled cargo loading into EVs, but also EV cargo mirroring features of parental cell.

In glioma, a study exploring EVs released by proneural, and mesenchymal glioma stem cells (GSCs) identified that proneural GSC-derived EVs lacked canonical EV-based tetraspanin markers such as CD9, CD63, and CD81, while they were abundant in mesenchymal GSC-derived EVs. Moreover, uptake of EVs derived from mesenchymal and proneural GSCs were differentially regulated in endothelial cells^43^. In a more functional context, glioblastoma cells were shown to secrete EVs capable of immunosuppression by blocking T-cell activation and receptor stimulation^44^. Specific packaging of Notch1 protein or VEGF-A in EVs enable GSCs to regulate other glioma cells or exert pro-angiogenic and immunosuppressive functions in the tumor niche^45,46^. High enrichment of specific ABC drug transporters in GSC-derived EVs have been shown to facilitate chemoresistance in glioblastoma^47^. These findings provide direct instances of EVs from different glioma cell states displaying source-specific heterogeneity in their cargo as well as functional nature.

## EV-mediated communication in glioblastoma heterogeneity

**Fig. 1:**
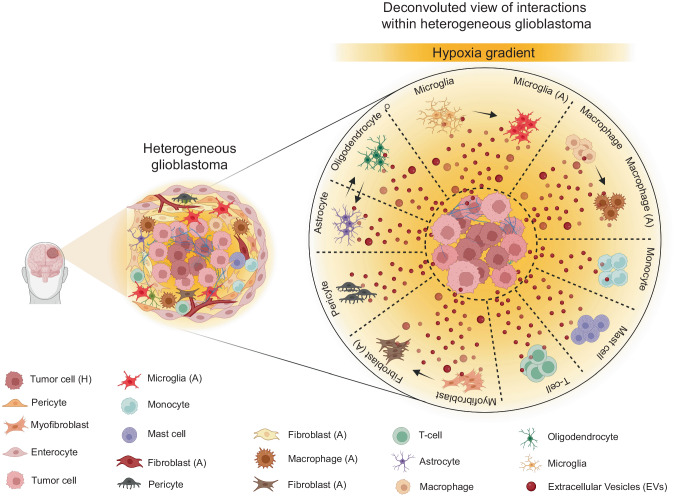
Schematic representation showing heterogeneous glioblastoma and a deconvoluted view of the heterogeneous interactions between tumor cells and other microenvironmental cells via extracellular vesicles (EVs). There is an EV-mediated bidirectional communication between tumor cells and their microenvironmental neighbors including pericytes, myofibroblasts, enterocytes, microglia, monocytes, mast cells, fibroblasts, macrophages, T-cells, astrocytes, and oligodendrocytes that reciprocally reprograms the functional nature of all cell types directly contributing to disease heterogeneity. EV-mediated interactions can also trigger a functional switch in tumor microenvironmental cells to an activated state, particularly in microglia and macrophages to both M1 and M2 phenotype, and in fibroblasts etc. Besides EV-mediated communication can enable lineage conversion to an aggressive phenotype, for instance, development of anaplastic astrocytoma from astrocytoma or transdifferentiation of an oligodendroglioma into anaplastic astrocytoma or intra-conversion of an astrocytoma into oligodendroglioma. Also, it is a well acknowledged fact that the EV-mediated interactions between cells within the tumor microenvironment niche are highly directional in nature, a phenomenon that has come to be attributed to the specific cargo carried by EVs. Besides, depending on the specific tumor microenvironment niches that cells reside, particularly cells residing in the hypoxic tumor regions release heterogeneous EVs with very distinct cargo and their concentration and size which are functionally and molecularly different from their immediate as well as physiologically normal counterparts. In diagram abbreviations; EVs (Extracellular Vesicles); (A) represents the activated state; and (H) represents the hypoxic state.

Interactions of tumor cells within TME via EVs have emerged as a critical factor contributing to glioblastoma heterogeneity^48^ (Fig. 1). The TME of glioblastoma comprises glioma cells, specialized GSCs, stromal cells including resident glial cells (oligodendrocytes, astrocytes, ependymal cells, microglia), and infiltrating immune cells such as monocytes, macrophages, and lymphocytes^49,50^. In glioblastoma, a bidirectional symbiotic communication between cells within the TME, through transfer of bioactive content in EVs, triggers dynamic changes via autocrine, paracrine, and endocrine routes^51^. In fact, initial findings of EVs ferrying specific oncogenic protein signatures associated with brain tumor^52^ provided unprecedented mechanistic understanding into their heterogeneity. For example, new insights were gained from the study showing horizontal transfer of *EGFRvIII* through EVs generating a more aggressive phenotype in recipient cells, thus providing a new mechanism for receptor tyrosine kinase heterogeneity in glioma^53^. Apart from the growth factor receptors, miRNA transfer via EVs play important roles in tumor heterogeneity, aggressiveness, recurrence, development of TME, and therapeutic resistance in glioma^54–56^.

Glioblastoma-relevant EVs were then discussed in context-specific roles of different EV subsets in the progression of glioma types^57^. In stem cells, intratumoral exchange of miRNAs has been shown to augment the heterogeneity of GSCs, reflecting on the highly heterogeneous glioblastoma subtypes^58^. Meanwhile, the TME stress condition, hypoxia or low oxygenation status, a hallmark feature of solid tumors is a critical driving force in glioblastoma intratumoral heterogeneity. Hypoxic region within the glioblastoma not only triggers multilevel modulation of genomic as well as secretome profiles^59,60^, but also cause hypoxic cells to release EVs with diverse profile that promotes angiogenesis, tumor vascularization, pericyte vessel coverage, cell proliferation^61,62^. Therefore, as glioblastoma progresses, highly directional and multifaceted EV-mediated interactions add further layers of heterogeneity to the primary tumor, which result in the propagation of an already existing tumor and establish multiple new lesions by preparing a heterogeneous tumor-promoting microenvironment. Recent publications are significantly reinforcing this context by studying heterogeneous glioblastoma-derived EVs that were shown to differ substantially from their normal glial counterparts in the cargo composition, supporting disease progression, relapse, immune evasion, and therapeutic resistance by creating a highly supportive TME^63^.

## EVs as a non-invasive liquid biopsy tool in glioblastoma

The wide range of factors, including EV-mediated communication implicated in glioblastoma heterogeneity diminishes hopes for the development of broadly applicable therapies and accurate diagnostic solutions. The complex heterogenous nature of glioblastoma places urgent emphasis on the need for more personalized approaches. Here, EVs reflecting cellular interactions within the glioblastoma TME provide a unique opportunity to be harnessed for the identification and constant monitoring of the disease status, while contributing to intra- and inter-patient heterogeneity. For this reason, EVs are increasingly being explored as a liquid biopsy tool for personalized diagnostic and therapeutic approaches and recent years have seen an exponential increase in related investigations^64^. EVs as liquid biopsy-based tools to non-invasively identify glioma status and heterogeneity were also comprehensively discussed in the recent RANO (Response Assessment in Neuro Oncology) working group assessment that also included Circulating Tumor Cells (CTCs) and Cell free Nucleic Acids (CfNAs)^65^. CTCs, CfNAs, and secreted proteins are also potential biomarker candidates, but their extremely demanding characterization and low abundance in biological fluids^66^, is a major drawback.

**Fig. 2:**
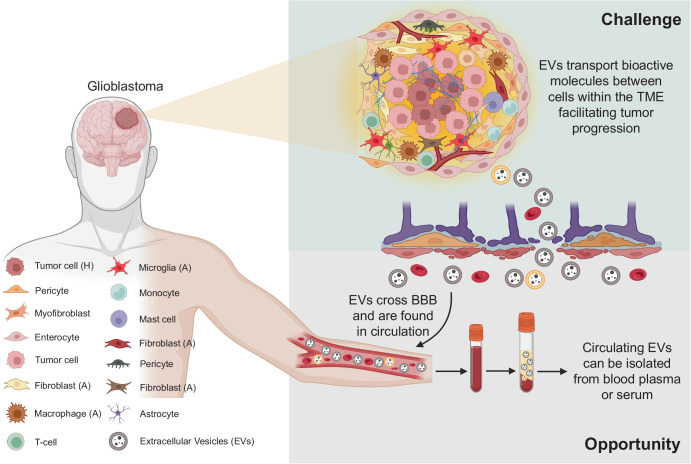
Schematic representation showing both the challenging scenario and opportunity presented by extracellular vesicles (EVs) in glioblastoma. EVs secreted by glioblastoma cells and other surrounding cells within the heterogeneous tumor microenvironment (TME) mediate communication and facilitate transport of pro-tumorigenic molecules between cells contributing to further tumor progression, which is a significant challenge. However, the EVs involved in intercellular communication within the glioblastoma microenvironment can cross the blood-brain barrier (BBB) and be found in systemic circulation. Circulating EVs can be isolated by suitable methodologies and profiled to understand the status and severity of the glioblastoma, providing a significant opportunity to utilize EVs both as biomarkers for disease diagnosis and to monitor response to treatment. In diagram abbreviations; EVs (Extracellular Vesicles); TME (Tumor microenvironment); BBB (blood-brain barrier); (A) represents the activated state; and (H) represents the hypoxic state.

The emergence of EVs as the most preferred liquid biopsy candidate primarily stems from their ability to mimic the pathological state of the host cell and interactions with the TME. In a pioneering set of studies, we found that EVs can reflect the hypoxic status of their host malignant glioma cells^36,61^. Since then, other studies have also substantiated the host-mimicking nature of EVs in glioblastoma^67^. This is particularly important from a clinical perspective as EVs can then be isolated from patient blood and profiled to understand the disease severity^68^ (Fig. 2). Meanwhile, EVs have a short terminal half-life that was found to be at the most 60 min^69–71^. From a diagnostic standpoint, when the short half-life of EVs is combined with their host mimicking properties, they can represent the prevalent biological and functional nature of the disease condition in real time^72^. This means EVs with unique source-specific identity, that is consistent with the disease status, can aid in accurate diagnostic discrimination as well as constant monitoring of the disease condition, making a strong case for their use in personalized approaches.

Additionally, EVs can cross the blood-brain barrier (BBB)^73^, but BBB selectively reduces the amount of detectable tumor-derived material in the blood stream^74^, which presumably applies to EVs as well. However, highly reliable and reproducible tools are now available to isolate a high yield of distinct EVs from biological fluids^75^. Systematic development of EV analysis tools including single vesicle measurements have ensured extraction of multilayered information from EV concentration and cargo on different grades, subtypes, molecular genotypes, and much of the microenvironmental phenotypic interactions within glioblastoma^74,76^ which is relevant for disease diagnosis and monitoring. Given these possibilities, there is a lot of interest in using EVs as source of biomarkers not only to study glioblastoma heterogeneity, but also to identify the disease severity.

## EVs for diagnosis and treatment response monitoring in glioblastoma patients – snapshot of actual disease progression

Accurate and timely diagnosis of glioblastoma is a critical necessity in prolonging the survival in patients. However, the suboptimal nature of current diagnostic tools^77^ and rising incidence rate^78^ has resulted in an increase in underdiagnosis of glioblastoma patients affecting their overall survival^79^. This is particularly prominent in low- and middle-income countries^80^. Invasive tissue biopsy followed by histological and molecular characterization fails to achieve a complete description of the whole tumor because of the heterogeneity and ineffective surgical sampling, and a need for longitudinal samples that may not always be available or accessible. Besides, tissue biopsy is also inherently limited in reflecting the temporal heterogeneity and longitudinal disease-related changes since surgical sampling is performed at a specific time point^76^. On the other hand, MRI cannot fully depict the current tumor status^81,82^ and more importantly, is unreliable to exclude pseudoprogression from actual tumor progression, that is found in more than 1/3rd of glioblastoma patients^82–84^.

**Fig. 3:**
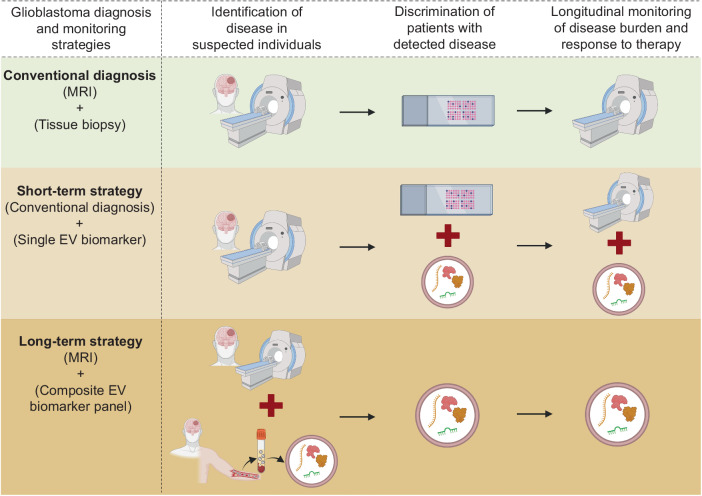
Schematic representation of currently used conventional diagnostic tools and prospective diagnostic strategies for glioblastoma identification, discrimination of patients with suspected disease into grades, and longitudinal monitoring of disease progression and/or response to therapy in postoperative and/or patients under therapy. In glioma diagnosis using conventional tools, Magnetic resonance imaging (MRI) is used to detect the tumor, followed by identification and discrimination of different tumor subtypes and grades by invasive tissue biopsy analysis. Then repeat MRIs are done to monitor the patient’s tumor progression and response to therapy. In the short-term strategy, after detection of tumor with MRI, tissue biopsy analysis can be combined with an extracellular vesicle (EV) biomarker to increase the accuracy of glioma subtype and grade identification. Further, combining EV biomarker with MRI can provide more reliability in longitudinal analysis of tumor progression and therapy response and overcome the limitations of MRI. In the long-term strategy, using a composite biomarker panel can either predict early disease or be combined with MRI for accurate disease detection. A composite biomarker panel that comprehensively describes the heterogeneous glioma can be used alone to identify the tumor subtype and grade, hence reducing, or even eliminating the need for invasive tissue biopsy analysis, as well as to monitor the disease progression and therapy response in postoperative patients. In diagram abbreviations; MRI (Magnetic Resonance Imaging); EV (Extracellular Vesicle).

In this scenario, developing a blood-based EV-associated non-invasive biomarker can be a reliable strategy to accurately identify glioblastoma severity. In fact, using well-defined clinical cohorts, we showed that EV-associated Syndecan-1 (EV SDC1) protein can identify high-grade glioblastoma from low-grade astrocytoma patients with high diagnostic accuracy. Moreover, in post-operative glioblastoma patients, EV SDC1 levels in blood plasma changed according to the extent of tumor resection indicating the reliability of EV-associated molecules for monitoring treatment response^85^. Furthermore, in a genome-wide methylation profiling study, DNA isolated from glioblastoma-derived EVs correctly identified their molecular classification^86^. Others studies have demonstrated that EV-associated molecules including mRNA^87^, circular RNAs^88^ and miRNAs^89,90^ can also be used for the discrimination of glioblastoma grades and subtypes and monitor dynamic molecular changes during tumor progression and therapy. This is indeed promising as EV-based biomarkers could then be explored to predict response of glioblastoma patients to prospective therapeutic agents as well as to exploit the maximum therapeutic potential of current immune checkpoint inhibitors which otherwise were less impressive due to the lack of accurate predictive biomarkers^91^. However, it is important to realize that one single protein or RNA-based biomarker associated with EVs might not reflect the true nature of glioblastoma heterogeneity, and hence may be inadequate for diagnostic discrimination and perhaps even monitor response to treatment. A short-term strategy could be to combine the single EV biomarker measurement with tissue biopsy analysis, that can increase the accuracy of disease diagnosis as well as improve the reliability of tissue biopsy analysis in clinical decision making. In the long-term, an EV-associated composite biomarker panel that comprehensively encompasses the changing molecular landscape of the heterogeneous glioblastoma would be a more reliable diagnostic tool to accurately identify glioblastoma as well as longitudinally monitor the disease progression and response to therapy. Moreover, the long-term strategy would provide additional clinical benefit of potentially reducing the number and frequency of life-threatening invasive tissue biopsies particularly for tumors that are inaccessible for surgical sampling (Fig. 3).

In the treatment sphere, one of the major limitations currently with therapy evaluation in glioblastoma is the lack of a biomarker that can accurately predict the clinical response. With treatment modalities causing pseudoprogression probably induced by local inflammation resulting in edema and increased abnormal vessel permeability at tumor sites^83,84^, constant monitoring of patients undergoing therapy by MRI is challenging. In the presence of these confounding factors, EVs carrying glioblastoma-associated proteins, mRNA, circular RNAs, or miRNAs that recapitulate the actual tumor progression as discussed earlier can be expected to better predict response to clinical interventions without requiring repeat biopsies.

## Concluding remarks

The heterogeneity within glioblastoma is a considerable challenge and if the increase in disease recurrence is any indication, the contribution of more variable factors cannot be ruled out. Though the role of EVs in glioblastoma heterogeneity only came to be appreciated after they covered some ground in other disease models, but now their contribution to glioblastoma progression is irrefutable. The decisive moment for EV research in glioblastoma came when it was revealed that they can mimic host pathophysiological features. Hence, EVs could provide insight into the heterogeneity of glioblastoma TME with considerable accuracy, that was only possible due to their direct role in cultivating such a pro-tumorigenic and immune suppressive microenvironment. This prompted researchers and clinicians alike to look at EVs as a potential source of biomarkers for disease identification. Given the significant limitations of conventional tools in glioblastoma, EVs that act as resources for tumor diagnosis and constant monitoring of dynamic molecular changes associated with disease progression may be the key to bridge the gap between theoretical and clinical implementation. The source-specific identity of EVs combined with their abundance in biofluids and easy characterization makes them a suitable liquid biopsy candidate for personalized approaches in glioblastoma diagnosis. It is important to note that over the last few years, EVs as a source of biomarker has also received increased attention across other tumor models, which further strengthens the clinical relevance of EVs as a liquid biopsy tool. In the future, to achieve the full diagnostic potential of EVs in glioma, more effort should be put on standardization of EV isolation from biofluids, choice of analyte detection strategies that can be scaled up, and design of clinical trials required for validation of these assays. In fact, these are some of the areas that have received considerable attention in recent years and led to the development of sophisticated tools in targeted profiling of EVs and scaling up for proteomic or genomic analysis in large batch of clinical samples. More findings in this direction are making a compelling rationale to revolutionize glioblastoma diagnosis and treatment through the implementation of EV-based liquid biopsy that allows consistent, noninvasive, and personalized assessment of tumor status and progression.

## Outstanding questions

- Can non-invasive biomarkers supplement current diagnostic tools? If relevant, can EV-based protein, RNA or other molecule measurements be combined with MRI and/or surgical tissue biopsy analysis data for more accurate and reliable detection of the glioblastoma condition?
- What is the significance of single biomarker versus composite biomarker panel in glioblastoma diagnosis and disease monitoring? Does a single EV-based biomarker fully represent the heterogeneity of glioblastoma despite demonstrating high diagnostic accuracy? If an EV-based composite biomarker panel has more utility, what combination of biomarkers would represent a consistent and reproducible approach in patients?
- Though the utility of EV-based biomarkers in personalized approaches means that they could address challenges posed by inter-patient heterogeneity, currently, very little information is available. Importantly, can EV-based biomarkers provide reliable diagnostic information in the event of unique tumor subclones present in patients with similar diagnostic classification?
- Currently, the correlation of biomarker measurements performed in blood plasma derived EVs to their pathological source is confirmed by analyzing the expression of the biomarker in tissue biopsy samples. In the future, one of the major challenges would be to develop a non-invasive tool that can aid in tracing the lineage of EVs from a particular source. Can an EV-based glioblastoma-specific signature be used to trace their lineage to brain tumor tissue?
- Presently, we cannot separate EVs in systemic circulation depending on their source of origin. This dilutes the significance of low abundant potential biomarkers measurements that are disease specific. Can an EV-based glioblastoma signature be used to enrich or sort brain tumor-derived EVs from systemic circulation? If so, what level of scaling up is needed to identify a disease-specific biomarker from tumor-derived EVs?
- Isolation of EVs from biofluids is not fully standardized, with different methodologies preferred for the characterization of different EV constituents. Therefore, to achieve full translational potential of EV-based biomarkers, can EV isolation from biofluids be simplified so that hospitals and other medical laboratories can perform them as part of routine clinical examination?
